# Exploring Students’ Use of Medical Education Resources for the USMLE Step 2 CK Exam Preparation

**DOI:** 10.1007/s40670-025-02362-3

**Published:** 2025-04-03

**Authors:** Brook A. Hubner, Zebulon Tolman, Omar Cherkaoui, Eileen Lee, Avery Stokes, Carolyn Klatt, Edward C. Klatt

**Affiliations:** 1 University of Alabama at Birmingham Marnix E. Heersink School of Medicine, 1720 2Nd Ave. S., Birmingham, AL 35294-3412 USA; 2 AMBOSS Inc, 144 W 27th St., New York, NY 10001 USA; 3 Mercer University School of Medicine, Mercer University Health Sciences Center, 1250 East 66th Street, Savannah, GA 31404 USA

**Keywords:** Medical student, Examination, Question bank, Resource usage, Study strategy, Learning method

## Abstract

**Introduction:**

The transition of the United States Medical Licensing Examination (USMLE®) Step 1 to pass/fail has increased the perceived importance of Step 2 Clinical Knowledge (CK) scores, making effective exam preparation increasingly important. Our study characterizes Step 2 CK resource usage patterns and analyzes their relationship with scores.

**Methods:**

Data from an anonymous, retrospective survey of fourth-year medical students from the Mercer University School of Medicine (MUSM) and the University of Alabama at Birmingham Marnix E. Heersink School of Medicine (HSOM) were analyzed to assess their study resource utilization patterns and self-reported Step 2 CK scores.

**Results:**

A total of 275 students reported average usage of 4.4 resources, including 1.6 question banks (Qbanks), 1.2 video, 0.6 podcast, 0.5 flashcard, and 0.6 print resources. All but one used Qbanks; all used digital resources, while 42.1% also used print resources. The mean Step 2 CK score of 247.3 (SD = 12.8) mirrored national averages. Total resources used did not impact scores significantly. Using 3 + Qbanks was significantly associated with lower scores. Video usage corresponded to lower scores. Students using no print resources scored significantly higher. Podcast and flashcard usage had no significant associations. The most popular resources were UWorld (97.7%), Anki (50.3%), Divine Intervention (50.3%), and AMBOSS (49.7%).

**Conclusion:**

Medical students accessed diverse resources for Step 2 CK preparation but focused on commercial question banks most frequently and print materials minimally. Students should avoid resource overload and emphasize active learning with 1–2 Qbanks and use other materials judiciously, building upon past course work to enhance Step 2 CK scores. Ongoing monitoring of study resource usage can guide a school’s academic coaching, curricular decisions, and resource acquisition.

**Supplementary Information:**

The online version contains supplementary material available at 10.1007/s40670-025-02362-3.

## Introduction

As the United States Medical Licensing Examination® (USMLE) Step 1 exam transitioned to pass/fail on January 26, 2022, USMLE Step 2 Clinical Knowledge (CK) emerged as the sole standardized, objective metric for evaluating applicant competitiveness, supplanting Step 1 scores as a key determinant for residency interview screening and selection into programs [1–4]. Students now face increased pressure to rigorously prepare for Step 2 CK to differentiate themselves amidst the uncertainty surrounding residency interview and rank order criteria determination. Medical educators and medical education learning specialists may need to expand their focus from primarily Step 1 preparation to robust support for Step 2 CK exam preparation [5–7].

Medical students have access to an abundance of electronic and print resources to support USMLE exam preparation and to supplement formal medical school curricular study material [8]. Students commonly exchange advice around preparation strategies and resource usage on social media forums, YouTube, TikTok, and other online platforms. In fact, on August 10, 2023, the #USMLEStep2 tag on TikTok had 24.7 million views. As crowdsourced recommendations, individual content creation, and commercial learning tools proliferate, medical educators and learning specialists are uniquely positioned to help students critically assess the breadth of formal and informal resources available for curricular and licensure exam support. Evidence-based advising drawing from student resource data learning science research can foster recommending strategic resource utilization. In effect, this approach shifts the focus from “what” to “how” and “why” resources should be used.

Retrieving information through practice questions, spaced-repetition activities, and early preparation have all been shown to improve Step 1 exam scores [9]. Spaced retrieval practice with repetition has been found to reinforce meaning, arrest forgetting, strengthen connections to prior knowledge, enhance retention, and bolster cues and retrieval [10]. Among these strategies, multiple studies highlight the considerable impact of exam practice questions on outcomes. Feedback from practice questions provide students with information about their strengths and weaknesses. Students who use this information strategically are more likely to achieve academic success because they are applying cognitive and metacognitive skills toward achieving learning goals. That is, they are engaging in self-regulated learning (SRL) by actively planning, executing, and monitoring performance through self-testing [11, 12].

SRL entails strategic and empowered learning processes. SRL is a proactive approach that moves beyond passively absorbing information to conscious application of a combination of cognitive, metacognitive, and motivational strategies to enhance learning experiences and achieve more meaningful and lasting knowledge acquisition [11, 12]. Cognitive strategies utilized to absorb and process information include utilizing mnemonic techniques, problem solving, and expanding upon and connecting information to existing knowledge and concepts [11, 12]. Motivational strategies involve sustaining enthusiasm and commitment to the learning process by finding personal relevance in the material, seeking challenges, and creating reward systems [11, 12]. A third essential component of SRL is the use of metacognitive strategies which refers to thinking about one’s own thinking [11, 12]. Learners with strong metacognitive skills are effective at planning, monitoring, and evaluating their learning process [10, 11].

The core of SRL is the idea that learners take ownership of their learning process. They do this by making decisions about their learning strategies, self-monitoring and recognizing when they are struggling, adapting their approach by seeking alternative resources or techniques, then reflecting and evaluating what worked well and what needs continued improvement [10, 11]. Successful students effectively utilize SRL strategies to plan resource utilization and assess progress by self-testing [13]. Medical students commonly utilize student-authored and commercial question banks (Qbanks) and crowdsourced Anki flashcard decks to self-test and monitor performance.

Consistent with research on Step 1 performance and a large body of evidence from the field of learning science, emerging literature points to practice questions as an optimal Step 2 CK preparation strategy. National Board of Medical Examiners (NBME) Comprehensive Clinical Science self-assessments have been found to be useful when assessing readiness for Step 2 CK [9, 14]. With Step 2 CK as one of the few standardized, objective metrics available to program directors in the residency application process, students, medical educators, and medical education learning specialists can benefit from an enhanced understanding of the resources utilized for Step 2 CK preparation. Using a lens of SRL theory, this study aimed to (1) characterize medical student use of educational resources for USMLE Step 2 CK preparation and (2) investigate the relationship between the total number and type of resources used and self-reported Step 2 CK scores at two medical schools.

## Methods

This study was approved by the UAB Institutional Review Board (Project ID 300009364) and MUSM Institutional Review Board (Project ID H2203078). The clinical trial registration is not applicable due to the nature of the research, which did not involve patient care. The study used a survey research design with a population of medical students at Mercer University School of Medicine (MUSM) and the University of Alabama at Birmingham Marnix E. Heersink School of Medicine (HSOM). The survey questionnaire was tested and developed by the authors. The survey was sent out electronically via email with web link between April 12, 2022, and December 9, 2022, via a web-based accessible platform developed by AMBOSS to medical students who had taken the USMLE Step 2 CK. Electronic informed consent was obtained using an approved online consent form before starting the survey and participants were able to terminate their participation at any time. Survey questions covered self-reported Medical College Admissions Test (MCAT), USMLE Step 1, and USMLE Step 2 CK scores along with usage patterns of study resources in preparation for the USMLE Step 2 CK. The anonymous data collected were analyzed by the authors.

After removing duplicate and ineligible responses, the adjusted response rates were 61% (*n* = 61) for MUSM and 71% (*n* = 110) for HSOM. Responses were flagged as duplicates if they had the same data entry for each of the following questions: “Step 2 CK exam date,” “Weeks dedicated to preparing Step 2 CK exam,” “MCAT score,” “Step 1 score,” and “Step 2 CK score.” When a duplicate response was identified, only the latter response was retained. A response was considered ineligible if it lacked a valid score for USMLE Step 2 CK exam (214–300).

Respondents reported their usage of study resources, including online Qbanks, videos, flashcards, podcasts, and print materials as either “Used for Step 2 CK only,” “Used for Shelf exams only,” “Used for Shelf exams and Step 2 CK,” “Did not use,” and “Never heard of it.” For this study covering resources used for Step 2 CK preparation, only respondents who answered “Used for Step 2 CK” or “Used for Shelf exams and Step 2 CK” were counted as having used the resource. Answering questions was voluntary, and respondents could opt out at any point. Respondents who opted out were not included. Non-responses for any single item in the survey were classified as “Did not use.” The HSOM questionnaire included an additional video resource called “Dr. High Yield.”

This study used a survey research design. One hundred fifty-five medical students from HSOM and 120 medical students from MUSM, all fourth year, were invited to participate in a survey focused on USMLE Step 2 CK resource usage. Both schools have a traditional 2-year preclinical and 2-year clinical curricula. The preclinical curriculum of each has integrated instructional content organized into basic science blocks of 3 to 6 weeks, beginning with fundamentals/foundations of medicine, followed by organ system blocks. The clinical years contain the standard clerkship rotations (family medicine, internal medicine, surgery, psychiatry, OB-GYN, pediatrics) in the third year, with a fourth year composed of selectives and electives. Eligible participants had completed USMLE Step 1 (before it became pass/fail), prior to beginning their third year of the curriculum, and completed Step 2 CK after completing their third year clerkships.

This study was approved by both institutions Institutional Review Boards. Respondents had the option to receive a $20 financial incentive. Incentive requests were submitted via a separate link, ensuring that identifying information was stored independently from study responses. Response rates were 61% (*n* = 61) and 71% (*n* = 110), respectively, and 171 surveys were analyzed. The survey comprised 27 multiple-choice and open-answer questions, 9 of which were pertinent to this study, about their use of medical education resources based on literature review [15–20], social media [21–25], and input from faculty. MUSM provides students with institutional access to UWorld and AMBOSS. HSOM did not provide institutional access. Neither school issues official USMLE preparation resource recommendations but shared commonly used resources identified through internal student usage surveys. The survey questions with explanatory programming methodologic markup for choices made are provided in Additional file 1.

Statistical analyses were performed in IBM SPSS 29.0. We summarized the descriptive statistics for total resources used, resource types, standardized exam scores, and dedicated study time. Bivariate cross-tabulations were then used to assess the potential impact of resource count, types, and dedicated study weeks on Step 2 CK scores. Next, we conducted a correlation analysis to explore relationships among standardized exam scores, dedicated study time, and total resource usage. Lastly, we performed one-way ANOVA analyses with post hoc tests to evaluate mean differences in Step 2 CK scores across total resources used, resource types, and standardized exam scores.

## Results

Survey respondents reported using an average of 4.4 resources for Step 2 CK preparation. Qbank, flashcard, and video resources were more commonly used than podcast and print resources. No statistically significant differences in overall resource utilization and mean Step 2 CK score were identified.

Qbank usage is reported in Table 1. Forty-eight percent of students reported using one Qbank, while 46.8% of students utilized 2 Qbanks. Eight students (4.7%) utilized 3 or more Qbanks. When comparing the differences in the number of Qbanks utilized and Step 2 CK score, a one-way ANOVA analysis with Tukey HSD post hoc tests (*F*_(2,167)_ = 3.635, *P* = 0.028) showed that students who utilized 3 or more Qbanks (M = 235.6, SD = 9.9) reported significantly lower mean (SD) Step 2 CK scores than students utilizing only 1 (*M* = 247.6, SD = 11.9) or 2 Qbanks (*M* = 248.2, SD = 13.5). There was no significant difference in Step 2 CK scores between respondents who used 1 or 2 Qbanks, although clear preferences stood out. UWorld was utilized by 98% of survey respondents and AMBOSS was utilized by 50% of survey respondents compared to USMLE-Rx (5%) and Kaplan (2%) Qbanks (Tables 2, 3, 4 and 5).

**Table 1:** Mean Step 2 CK score by total number of Qbanks

Qbanks	*n* (%)	Mean Step 2 CK Score (SD)
None	1 (0.6%)	253 (-)
1	82 (48.0%)	247.6 (11.9)
2	80 (46.8%)	248.2 (13.5)
3 or more	8 (4.7%)	235.6 (9.9)

**Table 2:** Mean Step 2 CK Score by total number of video resources

Video resources	*n* (%)	Mean Step 2 CK Score (SD)
None	69 (40.4%)	249.3 (11.0)
1	43 (25.1%)	249.0 (14.1)
2	31 (18.1%)	245.9 (14.3)
3 or more	16 (16.3%)	241.6 (11.7)

**Table 3:** Mean Step 2 CK Score by total number of podcast resources

Podcast resources	*n* (%)	Mean Step 2 CK Score (SD)
None	82 (48.0%)	246.0 (12.7)
1	82 (48.0%)	249.0 (12.9)
2 or more	7 (4.1%)	243.6 (10.7)

**Table 4:** Mean Step 2 CK score by total number of print resources

Print resources	*n* (%)	Mean Step 2 CK Score (SD)
0 print resources	99 (57.9%)	250.3 (10.8)
1 print resource	52 (30.4%)	243.7 (12.1)
2 + print resources	20 (8.2%)	242.0 (18.8)

**Table 5:** Summary of resources used for Step 2 CK exam preparation

Resources used	Resource type	*n* (%)	Mean Step 2 CK Score (SD)
UWorld	Qbank	167 (97.7%)	247.1 (12.7)
Divine Intervention	Podcast	86 (50.3%)	249.2 (12.3)
Anki	Flashcard	86 (50.3%)	249.3 (11.0)
AMBOSS	Qbank	85 (49.7%)	247.6 (14.1)
First Aid for the USMLE Step 2 CK	Print	59 (34.5%)	243.8 (14.0)
Sketchy Medical	Video	46 (26.9%)	246.2 (12.8)
Dr. High Yield	Video	44 (25.7%)	247.4 (12.1)
OnlineMedEd	Video	43 (25.1%)	241.7 (13.3)
Emma Holliday	Video	36 (21.1%)	244.3 (12.4)
Boards & Beyond	Video	21 (12.3%)	242.6 (17.6)
Step Up to Medicine	Print	20 (11.7%)	241.6 (18.3)
Osmosis	Video	11 (6.4%)	238.6 (8.8)
Lecture and school materials	Print	10 (5.8%)	250.5 (10.0)
USMLE-Rx	Qbank	9 (5.3%)	241.8 (9.8)
USMLE Step 2 Secrets	Podcast	6 (3.5%)	234.3 (13.8)
Ninja Nerd Lectures	Video	5 (2.9%)	242.4 (15.7)
Kaplan Qbank	Qbank	3 (1.8%)	236.7 (3.1)
BoardVitals	Qbank	3 (1.8%)	235.7 (11.6)
Case Files books	Print	2 (1.2%)	234.0 (5.7)

The following resources listed as choices in the survey had no reported usage: Print (Dr. Pestana’s Surgery Notes, Master the Boards series, Blueprints book series); Podcasts (The Curbsiders, The Clinical Problem Solvers, Core EM, Surgery 101, Emergency Medical Minute); Videos (Physeo, Lecturio)

Video usage is reported in Table 2. Sixty percent of respondents reported utilizing video resources. A one-way ANOVA analysis showed a significant difference in the number of video resources used and score (*F*_(3,167)_ = 2.925, *P* = 0.035). Tukey HSD post hoc tests showed that respondents who used 3 or more video resources had significantly lower scores on Step 2 CK (*M* = 241.6, SD = 11.7, *P* = 0.034) than those who used 0 video resources (*M* = 249.3, SD = 11.0). Sketchy Medical (27%), OnlineMedEd (25%), and the Dr. High Yield YouTube channel (26%) were the top video resources utilized (Table 5).

Podcast usage is reported in Table 3. Podcast usage was evenly split between those using no podcasts (*M* = 246.0, SD = 11.0) and those using 1 podcast (*M* = 249.0, SD = 14.1). A small group of students reported utilizing 2 or more podcasts (*M* = 243.6, SD = 10.7). The one-way ANOVA analysis found no significant differences in the number of podcasts used and Step 2 CK score (*F*(_2,168)_ = 1.471, *P* = 0.233). Divine Intervention was the top podcast choice, with 50% of respondents reporting using this resource (Table 5).

Print resource usage is reported in Table 4. Print resources were less heavily utilized than Qbanks, videos, and podcasts. The one-way ANOVA showed significant mean score differences based on print resource usage (*F*_(2,168)_ = 7.034, *P* < 0.001). The Tukey HSD pot hoc tests showed that the 58% of respondents who did not utilize print resources had a statistically significant higher mean score (*M* = 250.3, SD = 10.8,* P* < 0.05) compared to those who utilized 1 print resource (*M* = 243.7, SD = 12.1) and 2 or more print resources (*M* = 242.0, SD = 18.8). Scores did not differ significantly between those who used 1 print resource and 2 or more print resources (*M* = 242.0, SD = 18.8, *P* = 0.848). This analysis generated the highest *F*-value observed in this study, which suggests that using print resources accounts for the largest negative change in mean score. First Aid for the USMLE Step 2 CK was identified as the top print resource and utilized by 35% of respondents.

Table 5 summarizes resource usage and type with associated mean Step 2 CK scores, organized by reported usage. Anki, an open-source online program that utilizes spaced repetition with crowdsourced and individually created flashcards, was reported by 50% of respondents, tying with Divine Intervention as the second most utilized Step 2 CK preparation resource.

## Discussion

This study was designed to investigate the range of resources used by students to prepare for Step 2 CK and to explore relationships between resource usage and score. The popularity of Qbanks for Step 2 CK preparation is consistent with prior research regarding resource usage and academic performance, including Step 2 CK [27–34]. We noted a positive correlation between Qbank usage and increased mean scores. Similarly, the only reported flashcard resource, Anki, also had high usage. These findings align with educational research emphasizing the efficacy of active recall and spaced learning, facilitated by flashcards and Qbanks, in solidifying information and promoting learning [9, 26, 34–36]. Qbanks and flashcards also enable students to engage in self-testing to monitor progress, reinforcing their role as SRL tools for active learning and retrieval practice. The interleaving nature of these resources, where a broad range of topics can appear in each set of practice questions (especially when studied in random order), further promotes learning. Additionally, Qbanks and flashcards support the idea that creating desirable difficulty such as through random blocks of questions enhances learning by maximizing cognitive effort.

While our study supports the efficacy of Qbanks in Step 2 CK preparation, our findings highlight that more is not necessarily better. Specifically, using only one or two Qbanks significantly correlated with higher scores, while students using three or more Qbanks experienced lower scores. Those uncertain about their learning strategies and less responsive to internal feedback about their learning effectiveness may seek additional resources without a substantiated rationale. Students with poor SRL skills may neglect to critically evaluate their performance and fully leverage Qbanks through error analysis, targeted review of weak areas, and spaced, interleaved learning, which can hinder their progress and lead them to add yet another Qbank.

Students who reported higher mean Step 2 CK scores utilized fewer or no video resources. Similar to using three or more Qbanks, the significantly lower mean Step 2 CK scores among students using three or more video resources align with a recent study linking higher video consumption to lower Step 1 scores [37]. Watching educational videos is typically a passive study strategy, lacking effectiveness for learning and retention. Although learners with strong SRL skills might self-test, predict, and otherwise turn a primarily passive activity into active learning, the low mean scores for those reporting using three or more video resources likely point to students who do not employ strategic learning skills. Students who are not strong self-regulated learners may not utilize resources strategically and continue to look for “better” resources to improve.

Qbanks (UWorld and AMBOSS) and other electronic resources (Anki, Divine Intervention) that utilize principles of retrieval practice and spaced repetition were the most common preparation materials identified. There is a growing trend of students using fewer printed resources, and our study appears to align with this research [36]. While print resources were not highly utilized, those who reported utilizing print resources also reported significantly lower mean Step 2 CK scores. Although our study did not ask students to characterize how they used the resources, the results may indicate overreliance on passive learning. Podcasts might be considered another passive learning strategy; however, the most utilized podcast, Divine Intervention, utilizes a question-and-answer teaching modality. The problem-solving approach of Divine Intervention, where students can self-test to monitor their progress, may point to the popularity of this podcast.

Qbanks, videos, podcasts, print material, and related resources all have a place in Step 2 CK preparation strategies. Too often, students grapple with optimizing resources, relying on word-of-mouth without evidence-based recommendations. This study provides insight into commonly utilized Step 2 CK preparation resources. Outcomes indicate that Qbanks, flashcards, and a podcast that promotes retrieval practice are the most popular. Our results, in line with SRL, indicate that strategic use of a few resources appears to impact score outcomes. While this study focused on categorizing resource usage, future research is needed to establish the relationship between how resources are utilized and academic performance.

## Conclusions

A select few Step 2 CK resources, when strategically utilized and grounded in learning science principles such as retrieval practice, spaced repetition, and desirable difficulties, appear to positively impact score outcomes. Indiscriminately adding more resources does not appear to improve scores. Due to our study design and sample size, there was not sufficient statistical power to identify which specific resources were significantly associated with higher scores for Step 2 CK. Therefore, we recommend that medical educators and learning specialists focus on helping students identify the most suitable resources and how to use them effectively. Evidence-based guidance from educators and medical education learning specialists, informed by insights into resource usage and outcomes, can help students optimize their resource selection and performance.

Our study had several limitations. Step 2 CK scores were self-reported by participants; however, mean scores align with publicly reported score means by each participating institution. The survey did not list every resource, but respondents were given the flexibility to manually input any additional resources used. Our study was also limited by lack of insight into how resources were utilized and incorporated into study plans by individual students, which impacts the ability to interpret findings. One limitation of this study is the relatively small sample size, particularly in certain subgroups, which may reduce the statistical power of our analyses. This study was exploratory in nature, and the use of ANOVA was intended to identify potential trends rather than to confirm specific hypotheses. While post hoc comparisons help mitigate the likelihood of type I error, the results should be interpreted with some caution. Future research with a larger sample could further support these findings. Although students in the study cohorts were on clinical rotations during the COVID-19 pandemic, their dedicated preparation for Step 2 took place after lockdown restrictions were lifted and standard educational activities resumed.

## Supplementary Information

Below is the link to the electronic supplementary material.

Supplementary file1 (PDF 40 KB)

## Data Availability

The datasets generated during and/or analyzed during the current study are available from the corresponding author on reasonable request. The survey questionnaires along with datasets used and analyzed during the current study are available from the corresponding author on reasonable request.
