# Supplementary material for: Exploring Students’ Use of Medical Education Resources for the USMLE Step 2 CK Exam Preparation
Source: Med Sci Educ. 2025 Apr 3;35(3):1597–603. doi: 10.1007/s40670-025-02362-3 (PMC12228608; doi:10.1007/s40670-025-02362-3)
Supplement: Supplementary file 1 — Supplementary file1 (PDF 40 KB) [file 40670_2025_2362_MOESM1_ESM.pdf]

Medical Education Resources in the Curriculum Survey

1. Have you taken the Step 2 CK exam?
    a. Yes
    b. No //if selected No then terminate survey

2. When do you plan to take the Step 2 CK exam? (open-ended date) //show if "No" is selected then send to disqualify page

3. When did you take the Step 2 CK exam? (open-ended date) //show if "Yes" is selected

4. How many weeks did you dedicate solely to preparing for Step 2 CK? (open-ended number)

5. Please describe your use of these Qbank and flashcard resources for your clerkship year study prep:

Kaplan<br>UWorld<br>AMBOSS<br>USMLE Rx<br>BoardVitals<br>Anki<br>//randomize	Used for Shelf exams only (1)<br>Used for Step 2 CK only (2)<br>Used for Shelf and Step 2 CK (3)<br>Did not use(4)<br>Never heard of it (5)

6. Please describe your use of these digital and print resources for your clerkship year study prep:

First Aid<br>Master the Boards<br>Step-Up to Medicine<br>Dr. Pestana's Surgery Notes<br>Blueprints<br>Case Files<br>Your university's lecture and course material<br>//randomize	Used for Shelf exams only (1)<br>Used for Step 2 CK only (2)<br>Used for Shelf and Step 2 CK (3)<br>Did not use(4)<br>Never heard of it (5)

7. Please describe your use of these video and digital resources for your clerkship year study prep:

Boards & Beyond<br>OnlineMedEd<br>Physeo<br>Lecturio<br>Osmosis<br>Ninja Nerd Lectures<br>Emma Holliday<br>Sketchy<br>//randomize	Used for Shelf exams only (1)<br>Used for Step 2 CK only (2)<br>Used for Shelf and Step 2 CK (3)<br>Did not use(4)<br>Never heard of it (5)

8. Please describe your use of these podcasts for your clerkship year study prep:

Surgery 101<br>Divine Intervention<br>Emergency Medical Minute<br>The Curbsiders<br>Core EM<br>USMLE Step 2 Secrets/Inside the Boards<br>The Clinical Problem Solvers<br>//randomize	Used for Shelf exams only (1)<br>Used for Step 2 CK only (2)<br>Used for Shelf and Step 2 CK (3)<br>Did not use(4)<br>Never heard of it (5)

9. Would you please share your Step 2 CK exam score? (open-ended numeric response)
